# Supplemental Ascorbate Diminishes DNA Damage Yet Depletes Glutathione and Increases Acute Liver Failure in a Mouse Model of Hepatic Antioxidant System Disruption

**DOI:** 10.3390/antiox10030359

**Published:** 2021-02-27

**Authors:** Colin G. Miller, Jean A. Kundert, Justin R. Prigge, Julie A. Amato, Allison E. Perez, Lucia Coppo, Gabrielle N. Rizzo, Michael P. Kavanaugh, David J. Orlicky, Colin T. Shearn, Edward E. Schmidt

**Affiliations:** 1 Microbiology & Immunology, Montana State University, Bozeman, MT 59718, USA; colin.miller2@montana.edu (C.G.M.); jkundert@montana.edu (J.A.K.); justin.prigge@montana.edu (J.R.P.); allison.perez@student.montana.edu (A.E.P.); gabrielle.rizzo@student.montana.edu (G.N.R.); 2 Chemistry & Biochemistry, Montana State University, Bozeman, MT 59718, USA; 3 McLaughlin Research Institute, Great Falls, MT 59405, USA; julie@mri.montana.edu (J.A.A.); Michael.Kavanaugh@mso.umt.edu (M.P.K.); 4 Department of Medical Biochemistry & Biophysics, Karolinska Institutet, SE-171 77 Stockholm, Sweden; lucia.coppo@ki.se; 5 Department of Pathology, School of Medicine, University of Colorado Anschutz Medical Campus, Denver, CO 80045, USA; DAVID.ORLICKY@CUANSCHUTZ.EDU; 6 Department of Pediatrics, Gasteroenterology, Hepatology, and Nutrition, School of Medicine, University of Colorado Anschutz Medical Campus, Denver, CO 80045, USA; COLIN.SHEARN@CUANSCHUTZ.EDU

**Keywords:** ascorbate, glutathione, redox, NADPH, oxidative stress, thioredoxin, disulfide

## Abstract

Cellular oxidants are primarily managed by the thioredoxin reductase-1 (TrxR1)- and glutathione reductase (Gsr)-driven antioxidant systems. In mice having hepatocyte-specific co-disruption of TrxR1 and Gsr (TrxR1/Gsr-null livers), methionine catabolism sustains hepatic levels of reduced glutathione (GSH). Although most mice with TrxR1/Gsr-null livers exhibit long-term survival, ~25% die from spontaneous liver failure between 4- and 7-weeks of age. Here we tested whether liver failure was ameliorated by ascorbate supplementation. Following ascorbate, dehydroascorbate, or mock treatment, we assessed survival, liver histology, or hepatic redox markers including GSH and GSSG, redox enzyme activities, and oxidative damage markers. Unexpectedly, rather than providing protection, ascorbate (5 mg/mL, drinking water) increased the death-rate to 43%. In adults, ascorbate (4 mg/g × 3 days i.p.) caused hepatocyte necrosis and loss of hepatic GSH in TrxR1/Gsr-null livers but not in wildtype controls. Dehydroascorbate (0.3 mg/g i.p.) also depleted hepatic GSH in TrxR1/Gsr-null livers, whereas GSH levels were not significantly affected by either treatment in wildtype livers. Curiously, however, despite depleting GSH, ascorbate treatment diminished basal DNA damage and oxidative stress markers in TrxR1/Gsr-null livers. This suggests that, although ascorbate supplementation can prevent oxidative damage, it also can deplete GSH and compromise already stressed livers.

## 1. Introduction

Oxidative stress is defined as a substantial deviation from normal cellular redox steady state [1]. Cells generate reactive oxygen species (ROS) as a byproduct of cellular respiration and metabolism or in response to external factors such as xenobiotic exposure, radiation, or environmental pollution [2]. Inflammation also exposes surrounding cells and tissues to high levels of ROS. Hydrogen peroxide (H_2_O_2_), the major cellular oxidant, is generated from superoxide (O_2_^●−^) both spontaneously and catalytically by superoxide dismutases [3]. Superoxide is produced primarily by the mitochondria as a byproduct of cellular respiration and by other metabolic activities [4]. Some cytosolic and membrane-associated enzymes, including cytochrome p450s and NADPH-oxidases, also generate superoxide under certain conditions [5,6,7]. Other oxidants, including hypochlorous acid (HOCl), nitric oxide (^●^NO), hydroxyl radical (^●^OH), and singlet oxygen, can also be generated within cells or can accumulate in their environment [8]. These ROS have the ability to damage proteins, lipids, and nucleic acids. Oxidative damage has been implicated as contributing to numerous disease states including neurodegeneration, heart disease, and cancer [2].

Cells have potent endogenous antioxidant systems that play critical roles in both defense against ROS and repair of oxidative damage. Predominant among these are the two cytosolic disulfide reductase systems: one driven by thioredoxin reductase-1 (TrxR1) and one driven by glutathione reductase (Gsr) [9]. In the former system, reducing power (electron pairs) extracted from reduced nicotinamide adenine dinucleotide phosphate (NADPH) by the flavin-containing TrxR1 are used to reduce an active site disulfide bond in oxidized thioredoxin-1 (Trx1), generating reduced (dithiol) Trx1. In the later system, electron-pairs from NADPH are extracted by the flavin-containing Gsr and used to reduce glutathione-disulfide (GSSG) into two molecules of the thiol-containing tripeptide glutathione (GSH) [10]. Fueled by reducing power from the Trx1 and GSH systems, abundant cytosolic peroxiredoxins (Prx) and glutathione peroxidases (Gpx) rapidly reduce H_2_O_2_ and other hydroperoxides [11,12]. Ribonucleotide reductase uses this reducing power to generate DNA precursors for replication, genome repair, and mitochondrial replenishment [13]. Protein disulfides arising from ROS exposure are repaired back to thiols by Trx1, GSH, and the GSH-dependent glutaredoxins (Grx). Similarly, methionine (Met)-sulfoxides arising from ROS exposure are repaired by Trx1- or GSH/Grx-dependent Met-sulfoxide reductases (Msr) [14,15,16].

Combatting severe oxidative stress can consume considerable NADPH. To sustain an adequate supply, several regulatory systems, including direct oxidant-inhibition of a key glycolytic enzyme, glyceraldehyde phosphate dehydrogenase (GAPDH) and metabolic realignments by oxidant-responsive transcription factor (Nrf2) and signaling (5′ AMP-activated protein kinase, AMPK) pathways coordinately (1) re-prioritize glucose metabolism to favor NADPH production from NADP + over glycolysis; (2) suppress competing anabolic consumption of NADPH; and (3) induce NADPH-generating pathways [17]. In addition, since ROS generation is often a secondary consequence of exposure to electrophilic toxins, the oxidant-response pathways also activate drug metabolism “phase-2” conjugases, which glutathionylate, glucuronidate, or sulfate these toxins, as well as phase-3 transporters, which then export the conjugated toxins out of the cell [18,19,20].

In yeast or bacteria, genetic co-disruption of TrxR1 and Gsr is lethal [21,22]. Most mice with hepatic deletion of both TrxR1 and Gsr (TrxR1/Gsr-null), however, are long-term viable and exhibit relatively normal liver function. TrxR1/Gsr-null livers sustain redox homeostasis by an NADPH-independent system that uses catabolism of dietary methionine (Met), via the Met cycle and transsulfuration pathway, to generate Cys, which is then used for de novo GSH synthesis [9,23]. This GSH supports critical reduction reactions, generating GSSG, which is subsequently excreted from the cells [23]. Although this pathway is able to support survival, export of the oxidized GSSG makes this highly inefficient, and resting TrxR1/Gsr-null livers accumulate extensive protein and DNA damage [23,24]. Moreover, mice with TrxR1/Gsr-null livers experience a “crisis period” between 4- and 7-weeks of age, during which a subset of animals of both sexes die from acute liver failure [23]. The high levels of damage to liver macromolecules and the high frequencies of acute liver failure in these mice suggest that the balance between redox homeostasis and cell death is at the threshold at this critical period and is easily tipped toward failure.

A diverse group of dietary supplements are categorized as antioxidants. These nutrients have the potential to support or synergize with endogenous antioxidant systems, thereby bolstering the maintenance of cellular redox homeostasis. Mechanisms of antioxidant action are similarly broad. Some, like GSH, are true reductants that can provide the electrons to support the elimination of H_2_O_2_ or otherwise reduce oxidants or oxidized cellular molecules. Conversely, others, such as sulforaphane, are themselves non-hazardous electrophiles that induce cells to elicit robust cytoprotective gene expression responses [25]. Vitamin E (α-tocopherol) stably traps free radicals, thereby quenching free radical chain propagation [26,27]. N-acetylcysteine (NAC) is a potent antioxidant that can function in thiol-disulfide exchanges to liberate other thiols [28], provide a source of the limiting sulfur amino acid cysteine (Cys) for GSH biosynthesis during severe glutathionylation-induced GSH-depletion, or provide a source of intracellular hydrogen sulfide (H2S) which, in turn, can protect cellular thiols from overoxidation [29] or directly quench H_2_O_2_ [26]. Still others, including many complex natural products, might provide antioxidant support through mechanisms that remain unresolved [30]. One of the most potent antioxidants is ascorbate (Vitamin C), which is a true reductant, a potent free radical trap, and an essential cofactor for the generation of hydroxyproline by hydroxyl prolase [30,31,32,33,34]. Although nearly all plants and animals can synthesize ascorbate, humans cannot, making Vitamin C an essential nutrient for humans [33].

Importantly, all redox-active antioxidants are also pro-oxidants. Whereas their reduced state will have antioxidant activity, the utilization of this reducing power will generate the oxidized form of the compound, which will be a pro-oxidant. Examples include most thiol-containing antioxidants, such as GSH or Cys, which form disulfides during oxidation. These disulfides must, in turn, be reduced to dithiols by the cellular disulfide reductase systems, thereby consuming NADPH and disulfide reducing power. Another example is ascorbate, which oxidizes to dehydroascorbate (DHA). DHA, itself, is not active as an enzymatic cofactor, a free radical trap, or an antioxidant. DHA is also actively taken-up by hepatocytes and is thereafter reduced to ascorbate by GSH, Grxs, GSH-S-transferases (GST), or TrxR1 [33]. In this context, however, DHA is a pro-oxidant that requires the consumption of cellular NADPH for the subsequent reduction of GSSG to GSH or recycling of oxidized TrxR1 [35]. Importantly, since most extracellular fluids are oxidizing, many dietary antioxidants, such as GSH or ascorbate, will spontaneously oxidize into their pro-oxidant forms in circulation before they can enter cells, and therefore will require the consumption of NADPH and cytosolic disulfide reducing power before antioxidant benefits can be realized.

It remains unclear how well supplemental ascorbate can substitute for insufficiencies in the endogenous disulfide reductase systems. In one study, ascorbate supplementation in newborn rats was shown to compensate for GSH depletion caused by buthionine sulfoximine (BSO), which inhibits glutamate-cysteine ligase (Gcl), the first committed step in GSH biosynthesis. In that study, ascorbate supplementation resulted in both an increase in GSH levels and a decrease in mortality [36]. Additionally, in a child with a genetic deficiency in glutathione synthase (GS, the last step in GSH biosynthesis), administration of ascorbate resulted in increased plasma GSH levels [37]. The mechanisms underlying the ability of ascorbate to increase GSH levels in either of these reports, however, remain uncertain. Neither GSH nor GSSG can directly enter cells, and GSH, itself, cannot be synthesized without having both Gcl and GS activity [38]. This suggests that ascorbate could not have increased synthesis of GSH in these studies, but rather might have been able to re-prioritize residual GSH. In the current study, we tested whether ascorbate supplementation could decrease levels of hepatic oxidative stress and rates of acute liver failure in mice with TrxR1/Gsr-null livers. Results showed that, although ascorbate treatment did diminish DNA damage and levels of some oxidative stress markers, rather than either protecting these livers or favorably re-prioritizing hepatic GSH, ascorbate or DHA treatment caused hepatic GSH depletion, hepatocyte necrosis and increased spontaneous acute liver failure. These results highlight the ability of ascorbate supplements to have deleterious activities in some situations. In the absence of robust NADPH-dependent disulfide reductase systems, such as might occur during severe oxidative stress or exposure to metallic or organic electrophilic toxins or drugs, supplemental ascorbate might be a liability for liver health.

## 2. Materials and Methods

### 2.1. Mice, Supplementations and Harvests

Animal procedures were approved by the Montana State University (protocol numbers 2015-05, 2018-01, and 2021-118-01) or McLaughlin Research Institute (number 2017-ES/MK-23) Institutional Animal Care and Use Committees (IACUC). All mice used in this study were on a C57Bl/6J background. The *Gsr^null^* allele used in this study is a chemical mutagen-induced deletion that disrupts all protein-coding functions of the allele. Mice homozygous for this mutation (“Gsr-null”) are phenotypically normal and have been reported previously [39]. The *Txnrd1^cond^* allele is a targeted Cre-dependent conditional-null allele that encodes normal TrxR1 in the Cre-naïve state but is null in the Cre-recombined state, which has been described previously [40] and is available through Jackson Labs (Bar Harbor, ME, USA, JAX Stock #028283). Mice with TrxR1/Gsr-double-null livers are also whole body Gsr-null, but TrxR1-normal in all cell types except hepatocytes. These mice are overtly healthy as adults and are fertile in both sexes, although their livers show extensive chronic cell death, accumulation of oxidative damage, hepatomegaly, and hyperproliferation, as described previously [23,24,41]. All analyses shown used adult mice (60–90 days of age) of both sexes except as specified in the text or figure legends. Animals were maintained on a 14:10 h light:dark cycle with unrestricted access to feed and sterilized acidified water (pH 2.9–3.1, adjusted with HCl by a Hawkins automated doser, Hawkins, Roseville, MN, USA). Except as indicated otherwise, all harvests were performed between 9:00 and 11:30 a.m. For dietary supplementation, ascorbate was added to the acidified drinking water to a final concentration of 0.5% and this was replaced weekly; control animals received acidified drinking water without ascorbate. Importantly, the low pH of the acidified drinking water deters spontaneous oxidation of ascorbate to DHA [42].

### 2.2. Glutathione Assays

To measure GSH and GSSG levels from the same sample, snap-frozen liver pieces (~0.3 g) were homogenized in 0.8 mL of 10 mM HCl and proteins were removed by adding 5-sulfosalicylic acid to 1% (*w*/*v*) followed by centrifugation. Reaction mixes contained 120 mM NaH_2_PO_4_, pH 7.4, 5.3 mM EDTA, 0.75 mM DTNB (5,5′-dithiobis-2-nitrobenzoic acid; Sigma-Aldrich D8130, St. Louis, MO, USA), 0.24 mM NADPH and 1.2 IU/mL yeast Gsr (Sigma-Aldrich G9297), and 5 μL of a 1/20 dilution of clarified lysate, and were assayed at room temperature by absorbance at 412 nm in a Versamax plate reader (Molecular Devices, San Jose, CA, USA) [23,43]. Standard curves contained dilutions ranging from 0 to 1600 pmole GSH. To measure GSSG, 25 uL of the deproteinized lysate was added to 465 µL 120 mM NaH_2_PO_4_, pH 7.4, 5.3 mM EDTA and 10 µL 1 M 2-vinylpyridine in ethanol was immediately added. Samples were incubated at room temperature for 1–3 h in darkness to block free thiols. Assays used 20 µL of the blocked lysate in 120 mM NaH_2_PO_4_, pH 7.4, 5.3 mM EDTA, 0.75 mM DTNB, 0.24 mM NADPH and 1.2 IU/mL recombinant yeast Gsr (Sigma-Aldrich G9297). Standard curves contained 0–500 pmole GSSG and 20 mM 2-vinylpyridine. GSH concentrations were calculated by subtraction of GSSG concentration from total glutathione concentrations. Protein content was determined by the bicinchoninic acid (BCA) method following the manufacturer’s protocols (Sigma-Aldrich BCA1). In vivo redox probe imaging technologies have revealed that total glutathione pools in live cell cytosol are exceptionally reduced (GSH:GSSG ratios in cytosol of living cells ~10^4^) [44]. However, in biochemical assays, homogenization releases abundant GSSG from the endoplasmic reticulum (ER) [45], releases ROS from compartments including ER and peroxisomes, and exposes samples to environmental oxidants. As such, measured GSSG levels in biochemical analyses are typically orders of magnitude higher than the actual cytosolic level that was present in the pre-homogenized living cells or tissue (GSH:GSSG ratios typically 10^1^–10^2^). To assess how much GSSG in our samples arose from post-homogenization oxidation of GSH as opposed to release of compartmentalized GSSG, a separate GSSG assay was carried out wherein fresh-harvested liquid nitrogen-snap-frozen liver pieces and frozen buffer (containing 50 mM Tris pH 7.5, 150 mM NaCl, 1% NP-40, 50 mM *N*-ethylmaleimide, 30 mM iodoacetimide, 0.6% sulfosalicylic acid and 5% metaphosphoric acid) were co-pulverized into a fine homogeneous powder at −80 °C using a custom-fabricated ultra-low temperature Teflon/tungsten-carbide bead homogenizer (Imperium Engineering, Butte, MT, USA) driven by a B. Braun Melsungen Mikro-Dismembrator-II powerhead (Melsungen, Germany). The frozen powder was then thawed and incubated at room temperature to alkylate the GSH, followed by deproteination and dilution into the GSSG assay as described above. This procedure is not compatible with GSH measurements, so it was only used for GSSG validation. The results, shown in Supplementary Figure S3 had 2- to 3-fold less GSSG than did the non-alkylated samples (Figures 3–5 and Figure S1), yet also confirmed that total glutathione dynamics measured in this study were associated with loss of GSH and not substantial changes in the redox status of the glutathione pool.

### 2.3. Enzyme Activity Assays

Enzyme activities were determined for hepatic catalase (Cayman Assay Kit #707002, Cayman Scientific, Ann Arbor, MI, USA), superoxide dismutase (Cayman Assay Kit #706002), glutathione S-transferase (Cayman Assay Kit #703302) and glutathione peroxidase (Cayman Assay Kit #703102) following the instructions provided. TBARS (Cayman Assay Kit #10009055) were determined following manufacturer’s instructions.

### 2.4. Histological and Immunohistochemical Evaluation

Immunohistochemistry of control and 4-day Asc treated livers used the following antibodies and dilutions: rabbit polyclonal anti-pγH2A.X, 1:250 (Cell Signaling #9718, Cell Signaling Technologies, Danvers, MA, USA); rabbit polyclonal anti-4-hydroxynonenal (4-HNE), 1:500 [46]. Heat-induced antigen retrieval was performed in citrate buffer, pH 7.0, using a Biocare Decloaking System (Biocare Medical, Pacheco, CA, USA). Following overnight incubation with primary antibodies, slides were washed three times 5 min in tris-buffered saline + 1% tween and incubated in anti-rabbit-horse radish peroxidase (HRP)-conjugated secondary antibody for 30 min (Vector Labs, #MP-7401, Burlingame, CA, USA). The peroxidase substrate used was IMMPACT-DAB (Vector Labs, #SK-4105). Histologic images were captured on an Olympus BX51 microscope (Olympus-USA, Center Valley, PA, USA) equipped with a four-megapixel Macrofire digital camera (Optronics, Muskogee, OK, USA) using the PictureFrame Application 2.3 (Optronics). All pathology scoring was done on deidentified slides, such that the analysis was blinded to genotype, sex, and treatment conditions. For γ-H2aX scoring, hepatocytes with stained nuclei were counted on four to six images each from two to four slides from each animal captured at 200X magnification and an average score per frame was obtained. Values were divided by the average number of hepatocytes per 200X frame for each genotype, as indicated in the figure legend. Due to the universally weaker and non-nuclear nature of the 4-HNE staining in this study, seven frames shot at 100X magnification were imported into SlideBook (Intelligent Imaging Innovations, Denver, CO, USA) and the positive staining (pixels)/image was quantified.

### 2.5. Statistical Analyses

Statistical analyses were performed on Microsoft Excel 14.7 or Graphpad Prism 8.1 software. Bar graphs show means and SEM. Significance was determined by one-way ANOVA and post-ANOVA pairwise two-group comparisons with Tukey–Kramer method. Significance was assigned at *p* < 0.05. The significance of survival curves was calculated using log rank analysis.

## 3. Results

### 3.1. Ascorbate Supplementation Exacerbates Acute Liver Failure Frequencies in Mice with TrxR1/Gsr-Null Livers

**Figure 1:**
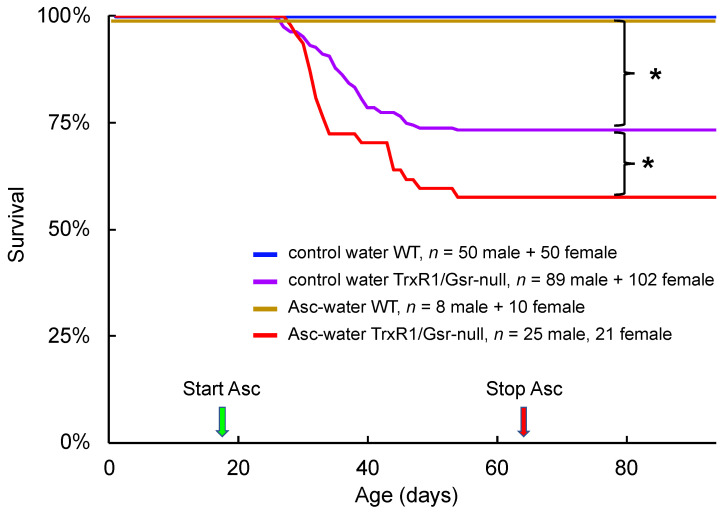
Impact of supplemental ascorbate on survival. Mice with WT or TrxR1/Gsr-null livers were raised on plain acidified drinking water (control) or acidified drinking water containing 5 mg/mL ascorbate (Asc-water) from weaning (P19, green arrow) until P63 (red arrow). The number and sex of animals represented is indicated; sexes did not differ significantly (*p* > 0.05) in any of the six groups (not shown). Brackets and asterisks, *p* ≤ 0.05, by log rank analysis.

Mice with TrxR1/Gsr-null livers are born at expected Mendelian frequencies; a portion of the animals of both sexes die of spontaneous acute liver failure between postnatal days 28–49 (P28–49), and animals surviving to P50 thereafter exhibit survival curves past P200 not differing substantially from those of matched WT mice [23]. Adult TrxR1/Gsr-null livers exhibit accumulation of damaged protein and DNA, high hepatocyte death indexes, and hyperproliferation [23,24]. Although it is not yet clear why liver failure is restricted to the three-week window of age from P28–49, the association with oxidative damage led us to hypothesize that liver failure results when individuals exceed a threshold of hepatocyte oxidative stress-induced cell death that becomes incompatible with liver survival and function. Therefore, we tested whether supplemental ascorbate could decrease the frequency of acute liver failure by ameliorating hepatic oxidative stress in these mice. Cages of pups having either TrxR1/Gsr-null (experimental mice) or WT (control) livers were placed on drinking water containing either 0% or 0.5% ascorbate at weaning (~P19) and maintained under these conditions until P63. Whereas the survival rate of animals with wildtype (WT) livers was 100% for mice on water with or without ascorbate, mice with TrxR1/Gsr-null livers exhibited ~25% lethality on water without ascorbate and, contrary to our expectations, this increased to 43% lethality with ascorbate supplementation (Figure 1). Pathological analyses of representative animals revealed that lethality was associated with extensive hepatocyte necrosis [23].

### 3.2. Ascorbate Causes Severe Pathology in TrxR1/Gsr-Null Livers

**Figure 2:**
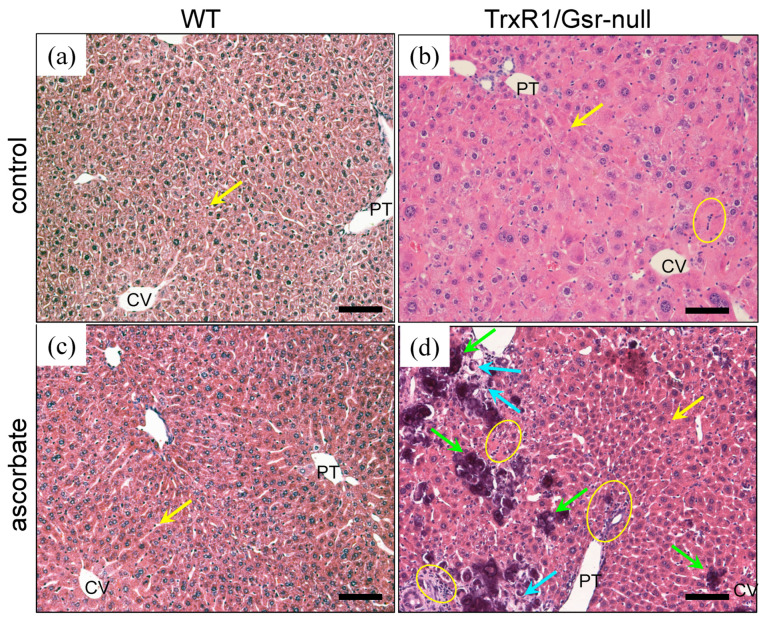
Histology of Gsr-null and TrxR1/Gsr-null livers treated with ascorbate. Mice with WT (**a**,**c**) or TrxR1/Gsr-null (**b**,**d**) livers received no treatment (control) or 4 daily i.p. injections of 4 mg/g ascorbate (ascorbate). Yellow arrows mark isolated sinusoidal leukocytes (small dark-staining cells) and yellow circles denote areas with larger groups of leukocytes. Green arrows denote intensely purple-staining Langhan-type giant macrophages, which tend to be focused in regions with increased necrotic hepatocytes (blue arrows). Representative images shown from *n* = 3–5 mice of each condition. CV and PT, representative central veins or portal triads, respectively. Scale bars, 100 µm.

To test whether ascorbate, itself, was compromising the TrxR1/Gsr-null livers, we administered ascorbate to resting adult mice (P60–90) via daily intraperitoneal (i.p.) injections at 0 (control) or 4 mg/g body weight in sterile saline. This is half the daily dose that had previously been shown under chronic administration to inhibit growth of oncogene-driven tumors without adversely affecting the mice, themselves [47]. Mice were sacrificed 3 h after the third daily inoculation and liver histology was examined. The histology of WT livers of either the control- or the ascorbate-treated mice showed no notable pathology (Figure 2a,c). By contrast, the livers of ascorbate-treated TrxR1/Gsr-null mice showed dramatic pathology (Figure 2d), which was substantially more severe than the basal pathology in untreated control TrxR1/Gsr-null livers (Figure 2b), as we had previously reported [23,24,41]. In many regions, hepatocytes in ascrbate-treated TrxR1/Gsr-null livers were necrotic, showed loss of cellularity, and had pyknotic nuclei (e.g., Figure 2d).

### 3.3. Ascorbate Treatment Depletes Glutathione in TrxR1/Gsr-Null but Not in WT Livers

**Figure 3:**
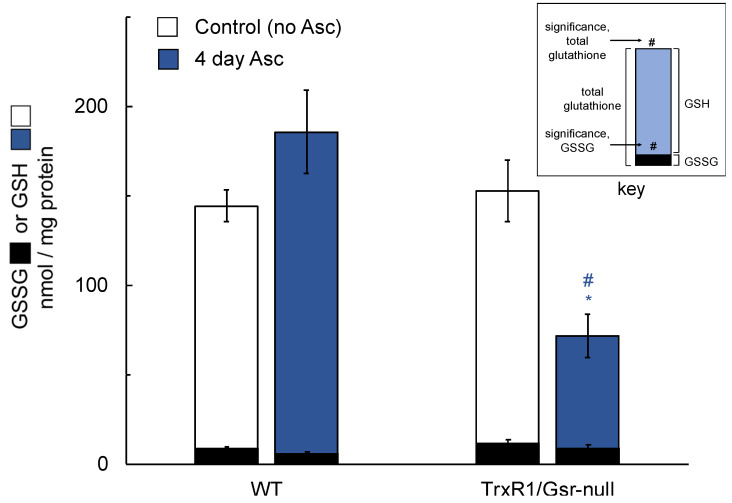
Hepatic levels of GSH and GSSG following 4-day ascorbate treatment. Mice received 0 (control) or 4 mg/g/day ascorbate × 4 days i.p. (Asc) and were harvested 3 h after the final inoculation. *n* ≥ 5 animals for each condition. Blue/white denotes GSH; black denotes corresponding GSSG in the same sample. Bars, mean ± s.e.m.; *, *p* ≤ 0.05 versus untreated control of same genotype; #, *p* ≤ 0.05 versus WT under same treatment using one-way ANOVA and Tukey–Kramer post hoc, pair-wise comparison.

**Figure 4:**
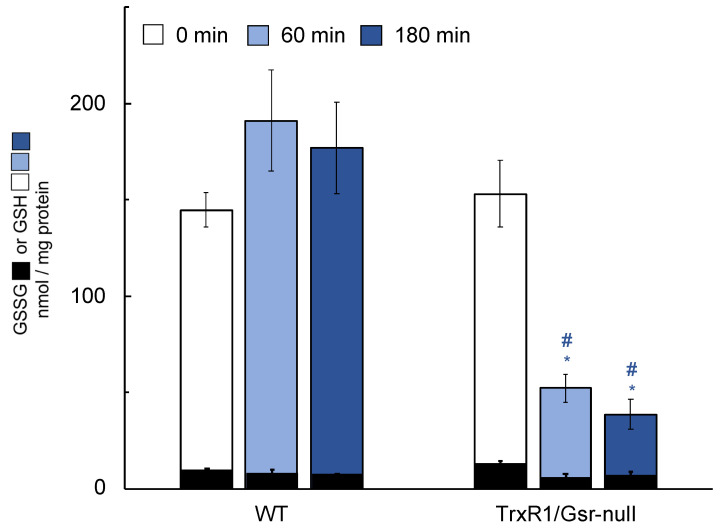
Hepatic levels of GSH and GSSG following short-term ascorbate treatment. Mice received a single dose of 4 mg/g ascorbate and were harvested at the indicated times thereafter (0 min mice received no ascorbate). *n* ≥ 5 animals for each condition. Green denotes GSH; black denotes corresponding GSSG in the same sample. Bars mean ± s.e.m.; *, *p* ≤ 0.05 versus untreated control of the same genotype; #, *p* ≤ 0.05 versus WT under same treatment using one-way ANOVA and Tukey–Kramer post hoc, pair-wise comparison.

The pathology induced by treatment of mice having TrxR1/Gsr-null livers with ascorbate suggested that ascorbate, itself, might be hepatotoxic in these severely reductase-compromised livers. Since these livers are highly sensitive to treatments that either block GSH biosynthesis or deplete glutathione stores [23], we investigated whether the ascorbate was impacting hepatic glutathione levels. As above, mice were given daily i.p. ascorbate injections and after day-4, livers were harvested and total glutathione (GSH + GSSG) or oxidized GSSG levels were measured in the liver lysates. Results indicated that daily ascorbate did not lower hepatic GSH + GSSG or GSSG in WT or Gsr-null livers; however, it significantly lowered GSH in TrxR1/Gsr-null livers (Figure 3). To determine whether loss of hepatic glutathione in ascorbate-treated TrxR1/Gsr-null livers occurred on a shorter timescale, mice were given a single dose of ascorbate in sterile saline (4 mg/g body weight) and were sacrificed 60 or 180 min later. No significant differences in glutathione were measured in WT livers 60 min after treatment with ascorbate (Figure 4). The TrxR1/Gsr-null livers had half as much total glutathione as untreated controls at 60 min, whereas the WT livers were unaffected. GSH depletion persisted at 180 min after injection in TrxR1/Gsr-null livers, yet WT GSH levels remained unaffected. Results indicated that administration of ascorbate led to hepatic glutathione depletion in livers lacking both NADPH-dependent disulfide reductases. Notably, the manifestation of this depletion (30% of initial levels after 4 d) was more modest than the rapid kinetics and large magnitude of the loss of hepatic glutathione we had previously measured upon administration of either BSO or acetaminophen to mice [23,24,48], in which nearly all glutathione was lost within 1 h of treatment. This suggests a distinct mechanism is driving glutathione depletion in response to ascorbate than those active in response to either BSO or acetaminophen (see below).

### 3.4. DHA Treatment Depletes Glutathione in Gsr, and TrxR1/Gsr-Null Livers

**Figure 5:**
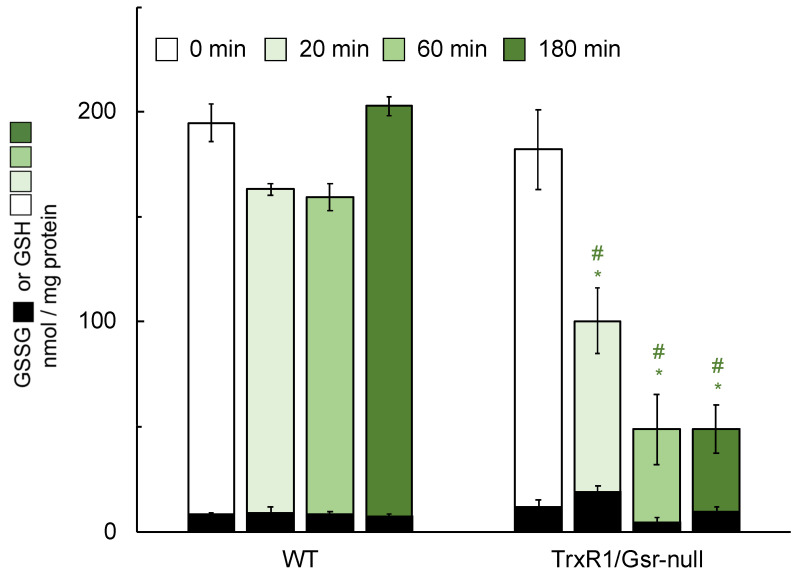
Hepatic levels of GSH and GSSG following short-term DHA treatment. Mice received a single dose of 0.3 mg/g DHA and were harvested at the indicated times thereafter (0 min mice received no DHA). *n* ≥ 5 animals for each condition. Green denotes GSH; black denotes corresponding GSSG in the same sample. Bars, mean ± s.e.m.; *, *p* ≤ 0.05 versus untreated control of same genotype; #, *p* ≤ 0.05 versus WT under same treatment using one-way ANOVA and Tukey–Kramer post hoc, pair-wise comparison.

The relatively modest impact of a single dose of ascorbate on hepatic glutathione levels (Figure 4) suggested that the observed glutathione depletion might have resulted from hepatic GSH-dependent reduction of DHA that was generated by spontaneous oxidation of the administered ascorbate after administration to the mice in the relatively oxidizing environment of the blood plasma and other extracellular fluids. To explore this possibility, mice were treated with DHA at 1/12th the dose (0.3 mg/g body weight) that we had used for ascorbate and were harvested at 0, 20, 60, and 180 min after inoculation. Results showed that DHA caused significant depletion of hepatic GSH in TrxR1/Gsr-null livers as early as 20 min post inoculation and, at 60 and 180 min, hepatic GSH levels were only 20% of the initial level (Figure 5). WT livers experienced no significant loss in hepatic GSH or increase of GSSG after DHA administration under the conditions used here. The possibility of a sex-specific difference in depletion of hepatic glutathione was investigated; however, no significant differences were measured between male and female mice (Supplementary Figure S1).

### 3.5. Ascorbate Treatment Lowers the Incidence of DNA Damage in TrxR1/Gsr-Null Livers

**Figure 6:**
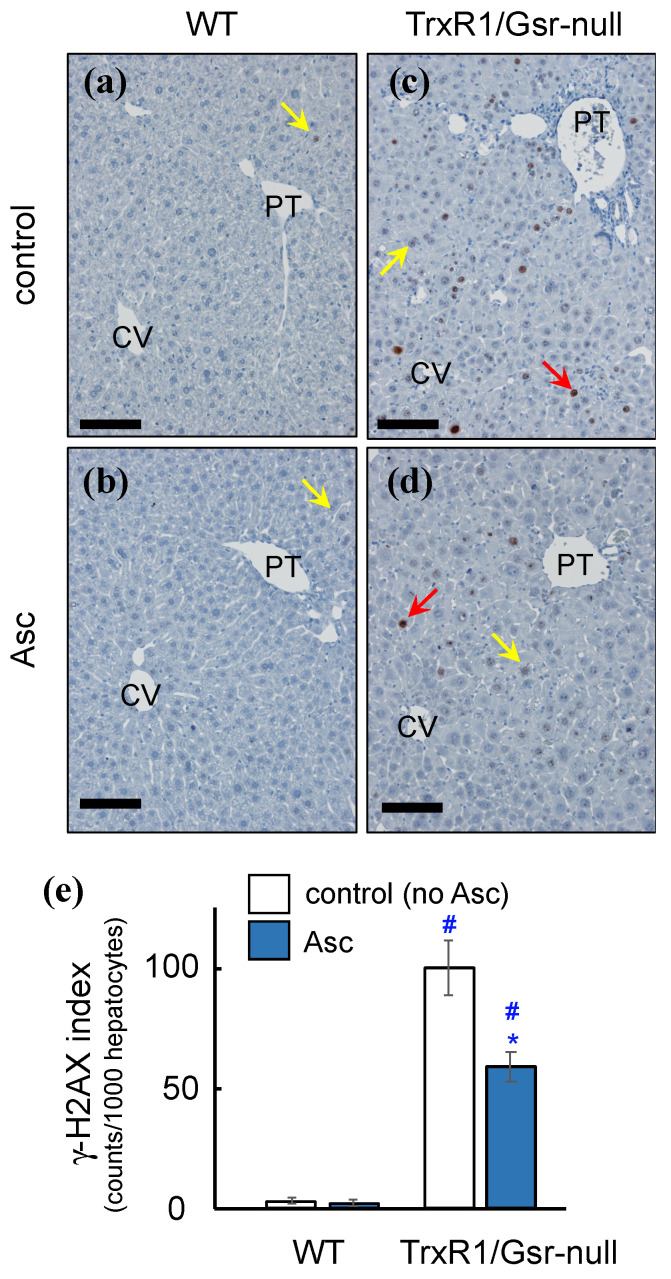
Impact of ascorbate treatment on basal levels of DNA damage. Mice received 0 (control) (**a**,**c**) or 4 mg/g/day ascorbate × 4 days i.p. (Asc) (**b**,**d**) and were harvested 3 h after the final inoculation. *n* = 3–5 animals for each condition; representative data shown. Liver sections were immunostained for γ-H2AX. Red and yellow arrows indicate representative strong- or weak-staining hepatocyte nuclei, respectively, all of which would have contributed to positive counts in the γ-H2AX index. CV and PT, central veins or portal triads, respectively. Scale bars 100 µm. Due to the previously reported genotype-specific cell size differences, photomicrographs at 200× magnification showed ~420 or ~285 hepatocytes per field of view for WT and TrxR1/Gsr-null livers, respectively. (**e**) Quantification of γ-H2AX staining index. Bars mean ± s.e.m.; *, *p* ≤ 0.05 versus untreated no-ascorbate control of same genotype; #, *p* ≤ 0.05 versus WT under same treatment using one-way ANOVA and Tukey–Kramer post hoc, pair-wise comparison.

Unlike WT or TrxR1-null, or Gsr-null livers, in which no evidence of basal oxidative damage is detected, TrxR1/Gsr-null livers exhibit dramatic oxidative damage [24,41,49]. The incidence of DNA damage is inferred by the presence of the phosphorylated form of histone protein H2AX (γ-H2AX), which is a chromatin modification that marks sites of double strand break repair. Immunostaining for γ-H2AX indicated that resting TrxR1/Gsr-null livers have a 50-fold higher incidence of γ-H2AX-positive hepatocytes than do WT livers (Figure 6). Interestingly, ascorbate supplementation (4 mg/g daily for 4 days i.p.) significantly lowered the γH2AX-staining index in TrxR1/Gsr-null livers (Figure 6c–e). This suggested that ascorbate was, indeed, functioning in an antioxidant capacity to diminish DNA damage (see Discussion).

### 3.6. Ascorbate did not Elevate Markers of Oxidative Stress in Liver

It was noteworthy that, in mice with Trxr1/Gsr-null livers, ascorbate treatment: (*i*) increased death-rates from acute liver failure (Figure 1); (*ii*) increased hepatic histopathology and the abundance of necrotic hepatocytes (Figure 2); (*iii*) decreased levels of hepatic GSH (Figure 3 and Figure 4); and yet (*iv*) also apparently protected the livers from DNA damage as compared to untreated mice with TrxR1/Gsr-null livers (Figure 6). Our anticipation would have been that the increased acute liver failure, histopathology, and hepatocyte necrosis were all directly caused by ascorbate treatment-induced oxidative damage that, in turn, resulted from the loss of GSH in the already disulfide reductase-deficient hepatocytes. However, the significant decrease in the γ-H2AX staining index was inconsistent with this and, instead, suggested that ascorbate treatment instead diminished oxidative damage in these livers. This, in turn, suggested that the pathology, necrosis, and acute liver failure in these animals were perhaps not driven by oxidative damage in the hepatocytes.

**Figure 7:**
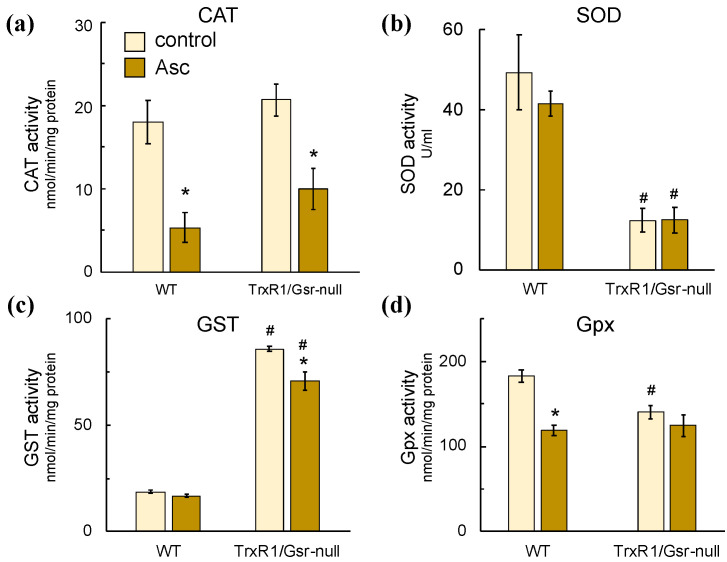
Enzymatic markers of oxidative stress. Enzyme activities of catalase (**a**, CAT), superoxide dismutase (**b**, SOD), GSH-S-Transferase (**c**, GST), and GSH-peroxidase (**d**, Gpx) were measured. Mice received 0 (control) or 4 mg/g/day ascorbate × 4 days i.p. (Asc) and were harvested 3 h after the final inoculation. *n* = 3–5 animals for each condition. Bars, mean ± s.e.m.; *, *p* ≤ 0.05 versus untreated no-ascorbate control of same genotype; #, *p* ≤ 0.05 versus WT using one-way ANOVA and Tukey–Kramer post hoc, pair-wise comparison.

To further investigate the redox status of the control and ascorbate-treated livers, we assessed other markers of hepatic oxidative stress. Measurements of lipid peroxide levels by the thiobarbituric acid-reactive species (TBARS) assay or of protein oxidative damage by immunostaining for either 4-hydroxynonenal or protein-glutathionylation revealed no significant differences between livers of any of the genotypes (Supplementary Figure S2 and reference [49]), indicating that the physiological outcomes do not include substantial lipid peroxidation or dramatic changes in protein damage. Next, we assessed levels of catalase, superoxide dismutase, glutathione-*S*-transferase, and glutathione peroxidase enzyme activities in the livers (Figure 7). WT livers treated with ascorbate for 4 days showed a roughly 3-fold decrease in catalase activity. Decreases in catalase activity were also measured in TrxR1/Gsr-null livers following ascorbate treatment; however, this only reached significance in the WT and TrxR1/Gsr-null livers (Figure 7a). Superoxide dismutase activity was roughly 4-fold lower in untreated TrxR1/Gsr-null livers, respectively, versus untreated WT livers; ascorbate significantly lowered superoxide dismutase activity in Gsr-null livers, but not in either WT or TrxR1/Gsr-null livers (Figure 7b). In contrast to superoxide dismutase, GST activity was dramatically elevated in TrxR1/Gsr-null livers, which is consistent with our previous reports showing an Nrf2-driven increase in GST mRNA, protein, and enzyme activity levels in these livers [41,48,49]; ascorbate treatment did not further increase GST activity in any genotypes and, instead, subtly but significantly decreased GST activity in TrxR1/Gsr-null livers (Figure 7c). Finally, although Gpx activity was subtly but significantly lower in untreated TrxR1/Gsr-null as compared to untreated WT livers, ascorbate treatment had no effect on Gpx activity in TrxR1/Gsr-null livers and caused a modest but significant decrease in Gpx activity in WT livers (Figure 7d).

## 4. Discussion

The rationale for people to use antioxidant supplements is to help support or bolster their endogenous antioxidant systems during oxidative stress. The efficacy of antioxidants has been demonstrated in diverse situations in cell culture models, animal models, and the clinic. Perhaps most dramatically, treatment of patients following exposure to hepatotoxic levels of acetaminophen with NAC prevents acute liver failure in ~70% of affected patients [50,51]. However, even in this well-studied situation, the exact mechanisms by which NAC promotes survival remain unclear, but clearly involve abrogation of hepatotoxic activities other than, or in addition to, oxidative stress. NAC, a simple *N*-acetylated version of Cys, undergoes complex and in many ways still mysterious extracellular and intracellular chemistry and metabolism following administration. The relative importance of its roles as a true reductant, a Cys-donor, a driver of thiol-disulfide exchange, or an H_2_S donor is not yet fully elucidated [28].

Vitamin C is perhaps the most commonly used antioxidant supplement. Its requirement as an essential micronutrient, its potency as a free radical trap, and its natural occurrence in many foods indicate it is safe and likely efficacious. As the non-enzymatic reaction of ascorbate with H_2_O_2_ is slow, and because mammals lack ascorbate peroxidase, ascorbate supplementation will not directly eliminate H_2_O_2_ [52,53]. Nonetheless, through its other activities, ascorbate is expected to support redox homeostasis in mammalian cells. The original goal of this project was to determine if dietary ascorbate supplementation could ameliorate the oxidative damage and risk of acute liver failure in the severely oxidatively stressed TrxR1/Gsr-null livers. Importantly, these livers are thought to model the effects of coincidental inhibition of TrxR1 and Gsr by strong electrophilic toxins [54], and as such might have value for testing therapeutic interventions for such conditions. As might be the case for many people considering ascorbate supplementation, we predicted that this supplement likely would be beneficial; we did not anticipate that it could possibly cause harm. The increased frequency of acute liver failure experienced with ascorbate was not expected.

We used i.p. inoculation of ascorbate to test whether the increased frequency of acute liver failure was, indeed, due to the ascorbate. We had previously shown that TrxR1/Gsr-null livers are exquisitely sensitive to treatments that deplete GSH [23,41,48], so we hypothesized that the hepatotoxic effects of ascorbate in these livers might result from GSH depletion. However, the modest and sluggish impacts that ascorbate inoculation had on GSH and GSSG levels in TrxR1/Gsr-null livers (Figure 3 and Figure 4) suggested the effect might be indirect and involve in vivo oxidation of the ascorbate to DHA. This prediction is consistent with the precipitous loss of GSH that occurred in livers of mice inoculated, instead, with a 12-fold lower dose of DHA (Figure 5).

In liver, DHA is reduced to ascorbate by GSH, Grxs, GSTs, and TrxR1 [33,55]. TrxR1 is reported to account for > 75% of the DHA reduction in rat liver cytosol [56]; however, since they lack TrxR1, TrxR1/Gsr-null livers must utilize GSH for all DHA reduction. Since these livers also lack Gsr, the resultant GSSG cannot be reduced. GSH/GSSG ratios are tightly regulated in the absence of Gsr by GSSG export [57], most likely by members of the ATP-dependent multidrug-resistance family of exporters (MDR or ABC proteins). Consistent with this, within the resolution that can be afforded by biochemical analyses, TrxR1/Gsr-null livers showed no increase in GSSG concentrations after ascorbate supplementation. It is also noteworthy in this regard that the TrxR1/Gsr-null livers exhibit very strong chronic activation of the Nrf2 pathway [41], and assessment of expression of several Nrf2 target genes suggests that ascorbate treatment has no substantial impact on the Nrf2 pathway in either WT or TrxR1/Gsr-null livers (Supplementary Figure S4). As TrxR1/Gsr-null livers are critically dependent on de novo synthesized GSH [23], the export of GSSG arising from GSH-dependent reduction of the supplement-generated DHA could lead to depletion of glutathione, as seen in this study. Nonetheless, the kinetics of depletion in the current study were slow and the magnitude was small compared to the precipitous crash seen in these livers following complete disruption of GSH biosynthesis with BSO or GST-mediated conjugation and export of GSH following high-dose acetaminophen challenge [23,58,59]. This suggests that GSSG export in the ascorbate-treated livers less drastically exceeded GSH biosynthesis capacity in this situation.

Like NAC, ascorbate undergoes complex chemistry and metabolism both extra- and intra-cellularly. In our survival study (Figure 1), the ascorbate was added to acidified drinking water (see Methods) to prevent its spontaneous oxidation [42] and this was refreshed weekly. However, even if oxidation was negligible in the water bottle, it could have been substantial after ingestion, in both the gut and the blood plasma. Similarly, when we administered ascorbate by i.p. inoculation, we minimized the likelihood of pre-administration oxidation to DHA, but not of in vivo oxidation. Indeed, we suspect that the slow and modest hepatic glutathione depletion caused by i.p. ascorbate (Figure 4) might, in part, reflect the rate of oxidation of the ascorbate to DHA in these animals and their consequential oxidation of hepatic GSH to restore the DHA to ascorbate. However, the short half-life of DHA in solution (~100 min) prevented a follow-up experiment supplementing DHA directly into the drinking water [60]. This instability also made direct analysis of DHA in animal samples impractical here.

At least two explanations might account for our observation that ascorbate treatment increased pathology and decreased GSH levels in the TrxR1/Gsr-null livers, yet also decreased evidence of DNA damage and other markers of oxidative stress. First, it is possible that the ascorbate, itself, interfered with the accuracy of some oxidative stress measurements. γ-H2AX staining is an indirect measure of DNA damage that might not be reliable during ascorbate treatment [61]. Additionally, ascorbate can interfere with catalase activity in vitro, likely through the required heme cofactor [62,63], although the timing (3 h post i.p. administration of ascorbate) and processing (perfusion before homogenization) in our protocol should leave little residual ascorbate. An alternative possibility, however, is that the pathology in the TrxR1/Gsr-null livers, both with and without ascorbate treatment, does not result from oxidative damage. Whereas oxidative damage is frequently cited as the ‘cause’ of cell death or disease in many situations, rarely is evidence shown to support causality over a correlative relationship. Recently, we showed that adult mouse livers coincidentally lacking TrxR1, Gsr, and Trx1 exhibit roughly an order of magnitude higher levels of γ-H2AX staining than do TrxR1/Gsr-null livers, as were used in the current study. This is matched with corresponding increases in other oxidative damage markers, yet the TrxR1/Gsr/Trx1-null livers remain functional and the mice with these livers exhibit long-term viability [41]. Such observations make us question whether oxidative damage, per se, is causing the pathology in many of the diseases that it correlates with. Rather than pathology via oxidative damage, we suspect that pathology results from a more general disruption to homeostasis. In the current study, the TrxR1/Gsr-null livers are already deficient in disulfide reducing power; the disulfide reducing power they obtain through catabolism of methionine is associated with re-prioritization of sulfur-, amino acid-, and energy-metabolism [9,23,64,65]. Ascorbate supplementation, even though measurably decreasing oxidative stress markers in these livers, collaterally consumes critical GSH, further stressing these metabolic pathways. The cause of necrosis or liver failure in these mice, therefore, might be hepatic amino acid or energy imbalances that disrupt translation, transcription, or other basal processes [65]. Ongoing studies are examining such mechanisms in the context of these genetically modified mouse liver models.

The important question arises: when is the administration of ascorbate safe and effective as an antioxidant, and when might it be dangerous? Clearly ascorbate, likely through in vivo oxidation to DHA, can consume hepatic GSH and disulfide reducing power. However, the ability of mice with TrxR1/Gsr-null livers to survive at all emphasizes that, in normal hepatocytes, the NADPH-fueled disulfide reductase systems can generate far more reducing power than is needed for survival [23]. We expect that cells in which these systems are robust will not be adversely affected by ascorbate supplementation, as noted for the control mice in the current study. Therefore, for combatting exposures that do not inhibit TrxR1 and Gsr, ascorbate supplementation could be beneficial. By contrast however, the current study shows that, when TrxR1 and Gsr are disrupted, as might occur with many electrophilic organic or metallic toxins or drugs [54], ascorbate supplementation could be detrimental. Consistent with this, the electrophilic metalloid arsenite is known to be a potent inhibitor of TrxR1 and likely also of Gsr [50,55,56]. Ascorbate supplementation was shown to increase lipid oxidation in cells challenged with arsenite [66]. Similarly, the reactive hepatic metabolite of acetaminophen, *N*-acetyl-*p*-benzoquinone imine (NAPQI), directly and potently inhibits TrxR1 and disrupts the GSH pathway [48]. Although acetaminophen overdose is associated with physiological responses in the liver that include oxidative damage [51], it might be prudent to caution against including ascorbate supplementation in therapeutic regimes to treat this condition. As in the mouse models presented here, ascorbate supplementation might contribute to GSH depletion in the NAPQI-challenged livers, and in the context of the metabolic stresses that these livers are already under, this could be harmful.

## Data Availability

The data presented in this manuscript are available from the corresponding author.

## Supplementary Materials

The following are available online at https://www.mdpi.com/2076-3921/10/3/359/s1, Figure S1, Sex- and genotype-specific differences in hepatic glutathione in mice. Figure S2, Genotype-specific accumulation of lipid peroxides and oxidative protein damage. Figure S3, GSSG levels measured under alkylating homogenization conditions. Figure S4, Effect of ascorbate treatment on Nrf2-response gene expression in liver.
