# Supplementary material for: Supplemental Ascorbate Diminishes DNA Damage Yet Depletes Glutathione and Increases Acute Liver Failure in a Mouse Model of Hepatic Antioxidant System Disruption
Source: Antioxidants (Basel). 2021 Feb 27;10(3):359. doi: 10.3390/antiox10030359 (PMC7997133; doi:10.3390/antiox10030359)
Supplement: Supplementary file 1 [file antioxidants-10-00359-s001.pdf]

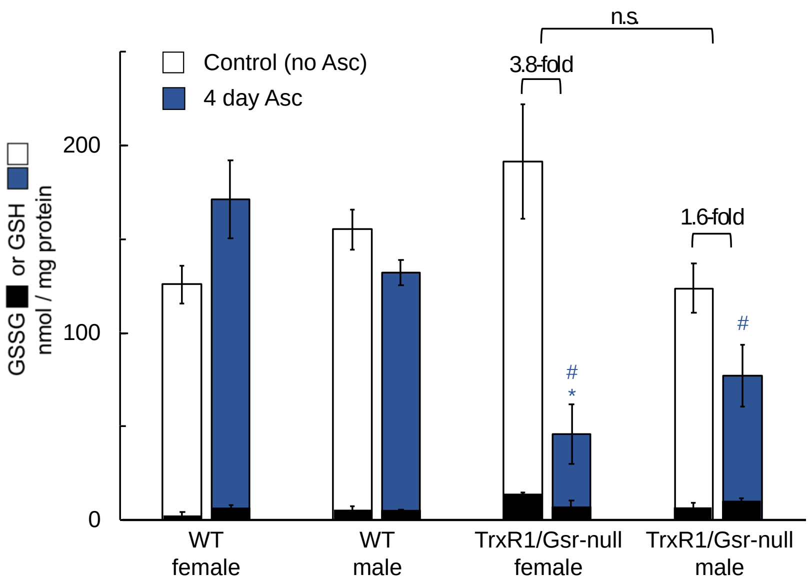

Figure S1. Sex-specific response of GSH and GSSG to ascorbate or dehydroascorbate treatment. Mice received 0 (control) or 4 mg/g/day ascorbate x 4 days i.p. (Asc) and were harvested 3 h after the final inoculation. *n* = 2–3 animals for each condition. Blue/white denotes GSH; black denotes corresponding GSSG in the same sample. Bars, mean ± s.e.m.; *, $p \leq 0.05$ versus untreated control of same sex and genotype; #, $p \leq 0.05$ versus WT of same sex under same treatment using one-way ANOVA and Tukey-Kramer post-hoc, pair-wise comparison. None of the values for total glutathione, GSSG, or GSH were significantly different between identically treated same-genotype samples of different sexes. n.s., the difference in GSH loss in response to ascorbate in TrxR1/Gsr-null female versus male livers was not significant, ($p \geq 0.05$).

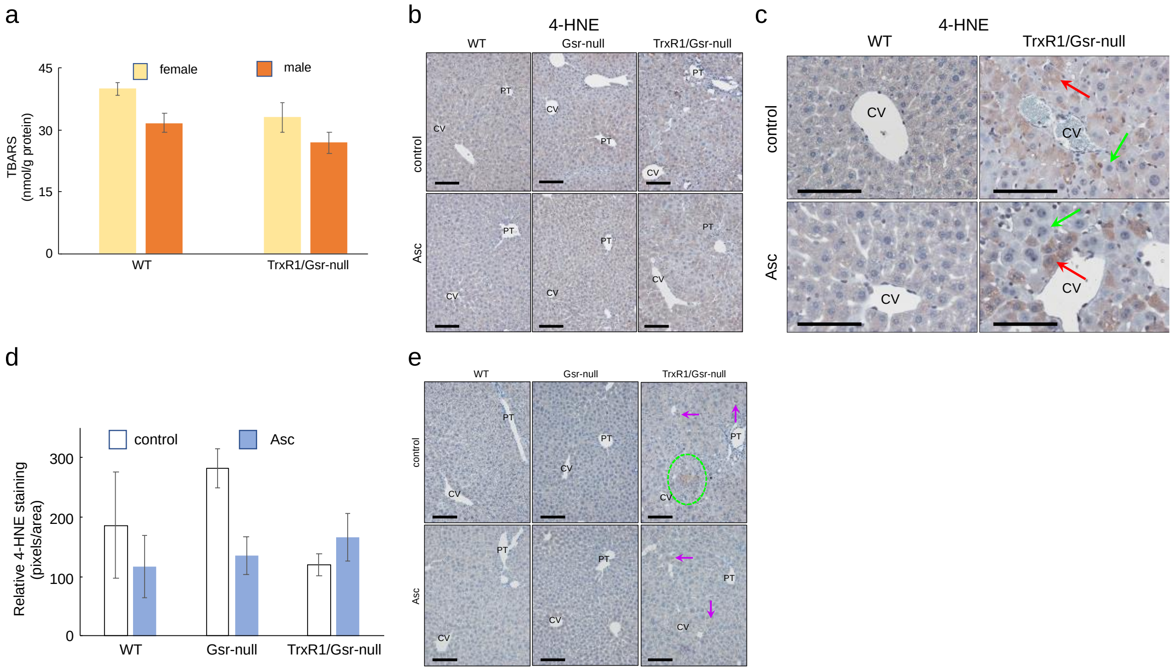

Figure S2. Accumulation of lipid peroxides, protein-4-HNE adducts, and protein-SSG adducts**.** (**a**), TBARS assay in male and female WT or TrxR1/Gsr-null livers reveals no significant differences between sexes or genotypes. *n* = 4–6-mice per experimental condition. (**b**), representative staining for 4-HNE in WT, Gsr-null, or TrxR1/Gsr-null livers either untreated (control) or treated for 4 d with daily 4 mg/g injections with ascorbate (Asc). CV, central vein; PT, portal triad. (**c**), higher magnification of 4-HNE staining in WT and TrxR1/Gsr-null livers as in (**b**) and (**d**) detailing the checkerboard distribution in the TrxR1/Gsr-null livers. Red arrows indicate 4-HNE-positive hepatocytes; green arrows indicate 4-HNE-negative

hepatocytes. d. Quantification of 4-HNE immunostaining on livers either untreated (control) or treated for 4 d with daily 4 mg/g injections with ascorbate (Asc). n = 3–6-mice per condition. e, Immunostaining for protein-SSG adducts. As reported previously [1], substantial protein-SSG was only detected in hepatocytes that appeared to be in the process of ballooning. Green dashed line circumscribes a region with hepatocytes stained for protein-SSG and exhibiting inflammatory cells (small dark-staining nuclei). Purple arrows denote non-hepatocyte cell types with protein-SSG staining. Scale bars 100 μm in all panels.

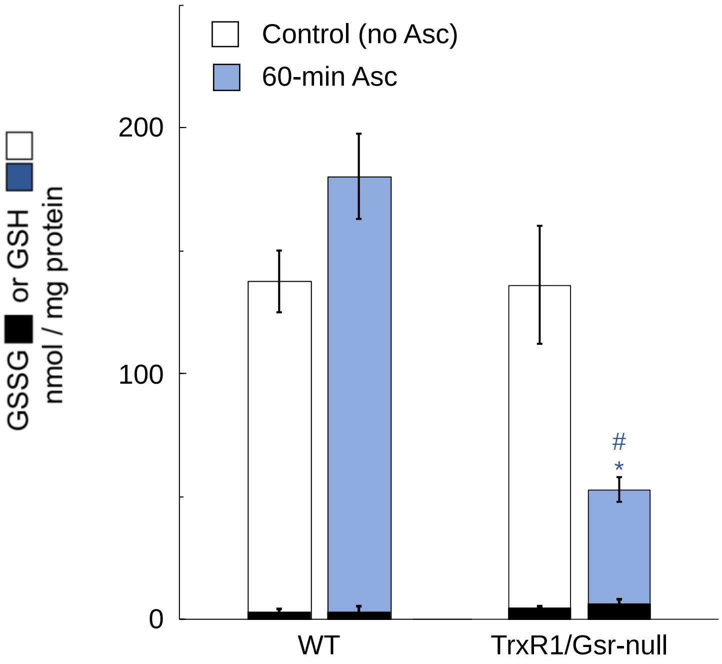

Figure S3. GSSG levels measured under alkylating homogenization conditions. Data presented in the manuscript measured both GSH and GSSG using the same liver homogenates, which were harvested under non-alkylating conditions to allow measurement of GSH. Sensitive live-cell fluorescent redox probes have revealed that GSH:GSSG ratios in the cytosol of living cells are ~10^4 [2]. In biochemical assays, however, apparent GSSG levels are inflated by (*i*) release of GSSG stores from the endoplasmic reticulum (ER)[3] during homogenization; (*ii*) release of ROS from the ER and peroxisomes during homogenization; and (*iii*) environmental ex vivo oxidation (discussed in the Materials and Methods section of main document). To determine the extent to which ex vivo oxidation was impacting GSSG values in this study, we cryo-homogenized livers in the presence of alkylating agents and then measured GSSG (black bars; see Materials and Methods section of main document). Since this precludes GSH analyses, the contributions of GSH shown (white/blue bars) are from separate lysates prepared by standard non-alkylating homogenization. Results showed that GSSG levels were ~2–3-fold lower in samples cryo-homogenized in the presence of alkylating agent but, importantly, GSSG levels did not differ significantly between any of the conditions nor account for differences in total glutathione levels. n = 6–7 biological replicates/assay. Bars mean ± s.e.m.; *, $p \leq 0.05$ versus untreated control of same genotype; #, $p \leq 0.05$ versus WT under same treatment using one-way ANOVA and Tukey-Kramer post-hoc, pair-wise comparison.

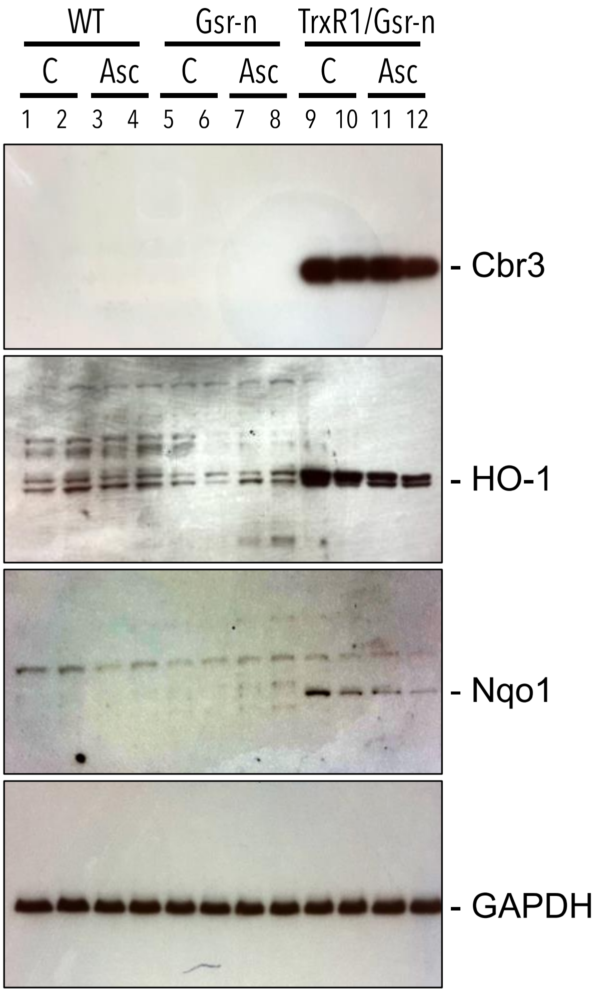

Figure S4. Effect of ascorbate treatment on expression of Nrf2-response genes in liver. Mice with wildtype (WT), Gsr-null (Gsr-n, not used elsewhere in this study), or TrxR1/Gsr-null (TrxR1/Gsr-n) livers received 0 (C) or 4 mg/g/day ascorbate x 4 days i.p. (Asc) and were harvested 3 h after the final inoculation. Total liver protein extracts were used for chemiluminescence western blot analyses with primary rabbit-antiserum raised against mouse carbonyl reductase 3 (Cbr3, antiserum kindly provided by GF Merrill, Oregon State University, 1:15,000 [4,5]), human heme oxygenase-1 (HO-1,Cell Signaling Technologies #D60G11,1:3,000), human NADPH-quinone oxidase-1 (Nqo1, Sigma-Aldrich #HPA007308, 1:500), and human glyceraldehyde-3-phosphate dehydrogenase (GAPDH, Proteintech #10494, 1:30,000 dilution). Cbr3, HO-1, and Nqo1 are representative Nrf2-respone genes [1]; GAPDH is a loading control.

Reference

1. M.R. McLoughlin, D.J. Orlicky, J.R. Prigge, P. Krishna, E.A. Talago, I.R. Cavigli, S. Eriksson, C.G. Miller, J.A. Kundert, V.I. Sayin, R.A. Sabol, J. Heinemann, L.O. Brandenberger, S.V. Iverson, B. Bothner, T. Papagiannakopoulos, C.T. Shearn, E.S.J. Arner, E.E. Schmidt, TrxR1, Gsr, and oxidative stress determine hepatocellular carcinoma malignancy, Proc Natl Acad Sci U S A 116(23) (2019) 11408-11417.
2. M. Gutscher, A.L. Pauleau, L. Marty, T. Brach, G.H. Wabnitz, Y. Samstag, A.J. Meyer, T.P. Dick, Real-time imaging of the intracellular glutathione redox potential, Nature methods 5(6) (2008) 553-9.
3. J. Birk, M. Meyer, I. Aller, H.G. Hansen, A. Odermatt, T.P. Dick, A.J. Meyer, C. Appenzeller-Herzog, Endoplasmic reticulum: reduced and oxidized glutathione revisited, J Cell Sci 126(Pt 7) (2013) 1604-17.
4. D.H. Breysse, R.M. Boone, C.M. Long, M.E. Merrill, C.M. Schaupp, C.C. White, T.J. Kavanagh, E.E. Schmidt, G.F. Merrill, Carbonyl Reductase 1 Plays a Significant Role in Converting Doxorubicin to Cardiotoxic Doxorubicinol in Mouse Liver, but the Majority of the Doxorubicinol-Forming Activity Remains Unidentified, Drug Metab Dispos 48(3) (2020) 187-197.
5. C.T. Shearn, B. Fennimore, D.J. Orlicky, Y.R. Gao, L.M. Saba, K.D. Battista, S. Aivazidis, M. Assiri, P.S. Harris, C. Michel, G.F. Merrill, E.E. Schmidt, S.P. Colgan, D.R. Petersen, Cholestatic liver disease results increased production of reactive aldehydes and an atypical periportal hepatic antioxidant response, Free Radic Biol Med 143 (2019) 101-114.
